# Longitudinal genome-wide methylation study of Roux-en-Y gastric bypass patients reveals novel CpG sites associated with essential hypertension

**DOI:** 10.1186/s12920-016-0180-y

**Published:** 2016-04-22

**Authors:** Adrian E. Boström, Jessica Mwinyi, Sarah Voisin, Wenting Wu, Bernd Schultes, Kang Zhang, Helgi B. Schiöth

**Affiliations:** Department of Neuroscience, Functional Pharmacology, Uppsala University, BMC, Box 593, 751 24 Uppsala, Sweden; Institute for Genomic Medicine, University of California, San Diego, CA 92093 USA; eSwiss Medical and Surgical Center, St Gallen, Switzerland

**Keywords:** Essential hypertension, Epigenome-wide association study, Whole methylome analysis, Bariatric surgery, *EHMT2*, *FUSSEL18*, *SKOR2*, *Corl2*

## Abstract

**Background:**

Essential hypertension is a significant risk factor for cardiovascular diseases. Emerging research suggests a role of DNA methylation in blood pressure physiology. We aimed to investigate epigenetic associations of promoter related CpG sites to essential hypertension in a genome-wide methylation approach.

**Methods:**

The genome-wide methylation pattern in whole blood was measured in 11 obese patients before and six months after Roux-en-Y gastric bypass surgery using the Illumina 450 k beadchip. CpG sites located within 1500 bp of the transcriptional start site of adjacent genes were included in our study, resulting in 124 199 probes investigated in the subsequent analysis. Percent changes in methylation states and SBP measured before and six months after surgery were calculated. These parameters were correlated to each other using the Spearman’s rank correlation method (Edgeworth series approximation). To further investigate the detected relationship between candidate CpG sites and systolic blood pressure levels, binary logistic regression analyses were performed in a larger and independent cohort of 539 individuals aged 19–101 years to elucidate a relationship between EH and the methylation state in candidate CpG sites.

**Results:**

We identified 24 promoter associated CpG sites that correlated with change in SBP after RYGB surgery (*p* < 10^−16^). Two of these CpG loci (cg00875989, cg09134341) were significantly hypomethylated in dependency of EH (*p* < 10^−03^). These results were independent of age, BMI, ethnicity and sex.

**Conclusions:**

The identification of these novel CpG sites may contribute to a further understanding of the epigenetic regulatory mechanisms underlying the development of essential hypertension.

## Background

Hypertension (HT) is the leading risk factor for mortality worldwide and is ranked third as a cause of disability-adjusted life-years. Today, nearly one billion people suffer from HT, making it an important public-health issue [1]. The high prevalence of HT and its identification as a major risk factor for secondarily occurring diseases of the cardiovascular system and the kidneys underlines the necessity to study the pathophysiological mechanisms and molecular biological susceptibility factors underlying the development of this disease.

The heritability of HT is estimated to range between 40 and 60 % [2]. Besides the well-established link between genetic and non-genetic factors (such as e.g. obesity, alcohol or salt intake) in the pathogenesis of HT, there is increasing evidence that epigenetic mechanisms might be able to contribute to the development of HT [3]. Especially the missing heritability conundrum of HT combined with its late onset and progressive nature point to epigenetics as a potential modulator of blood pressure (BP). Epigenetic patterns are potentially reversible and are influenced by nutritional-environmental factors as well as gene-environment interactions. These factors have been shown to play an important role in the development of complex multifactorial diseases, such as those affecting the cardiovascular system [3]. An increasing number of studies suggest a significant role of epigenetic modifications in the progression of EH [4–6].

An elementary pillar in antihypertensive therapy is the reduction of weight. It has been shown that a modest weight loss by 10 % already lowers the BP significantly [7, 8]. Surgical interventions such as Roux-en-Y gastric bypass (RYGB) surgery are the most efficient therapeutic options for a significant reduction of weight and an improvement of associated cardiovascular and metabolic co-morbidities today [9]. The reduction in BP typically seen after surgery has been suggested to be an effect of both caloric restriction and weight loss [10, 11].

Age significantly impacts the pathogenesis of EH [12] and different sets of genes control BP regulation at different ages [13]. Furthermore, men have a significantly higher risk to develop cardiovascular disease than age-matched premenopausal women [14]. In addition to disease risk alterations due to age, body weight and gender, the prevalence of EH varies in dependency of ethnic origin. So show individuals of Hispanic origin significantly lower BP levels than white individuals [15]. Inflammatory processes have also been shown to play a significant role in the pathophysiology of EH, whereby the relative contribution of inflammation to BP variability remains to be fully clarified [16].

To date, very few studies have investigated the association between DNA methylation and BP levels in a genome-wide study approach. In this study, we perform a whole methylome analysis in obese patients undergoing RYGB surgery to examine whether and to what extent changes in DNA methylation patterns are associated with changes in systolic blood pressure (SBP). Newly identified candidate CpG loci are further investigated and confirmed as risk loci for EH in a targeted analysis performed in a large cohort of 539 individuals.

## Methods

### Characterization of the discovery group and validation cohort

#### Study population of the discovery dataset

Samples used in this study have been collected in the frame of a study that has been previously published [17]. The study population included twelve obese patients, who underwent bariatric surgery at the Klinik Stephanshorn in St. Gallen, in 2009–2011. All subjects were eligible for bariatric surgery by international guidelines [18] and gave their written informed consent to participating in the study. In 2008 and prior to enrollment, the canton St. Gallen ethics committee approved the study.

Subjects were fasting at the time of blood sampling for whole methylome determination and measurements. Blood was drawn according to standard procedures and BP was measured one time after an overnight fast, in a sitting position, after 5 min of relaxation, before and six months after surgery. One patient suffered from a known genetic disorder and was not eligible for further analysis, resulting in a sample size of 11 individuals.

The surgical procedure was performed by connecting a 20–30 ml large gastric pouch from the upper stomach to the proximal jejunum, thereby considerably limiting the gastric residence time of nutrients. Furthermore, an anastomosis of the biliopancreatic limb to the jejunum was established 150 cm distal from the pouch-jejunal connection.

#### Study population of the validation dataset

Data for the validation study cohort were obtained from publicly available sources (ArrayExpress databases, accession number E-GEOD-40279). This cohort was composed of two different ethnic groups, comprising 426 Caucasians and 230 individuals of Mexican origin, aged 19 to 101 years old [19]. BMI was measured for all subjects. Information on blood pressure status (EH or normotensive) was provided to us by Zhang et al., and was available for 540 individuals. Information on intake of any anti-hypertensive medications, DBP or SBP, was not available. One patient was described as having ‘borderline’ EH, and was therefore excluded in our analysis, resulting in a sample size of 539 observations.

### Methylation profiling

Extraction of genomic DNA was performed by the phenol-chloroform method [20], after which it was bisulfite converted using the EZ DNA Methylation – GoldTM kit (ZymoResearch, USA). The bisulfite converted DNA was hybridized to the Illumina 450 k methylation chip (Illumina, San Diego, CA, USA) to measure DNA methylation in 485 577 individual CpG sites. In the discovery group, DNA obtained from blood samples taken from the same individual before and six months after surgery were hybridized to the same physical chip. The array was imaged using the Illumina iScan system (Illumina, San Diego, CA, USA), determining the percentile methylation for each CpG site across the study groups.

#### CpG site annotation

We used the expanded annotation table provided by Price et al. [21] to define the associated gene, distance to the closest TSS and the surrounding CpG density for each investigated CpG site. Probes are located in either a high-density islands (HC), intermediate-density islands (IC) or in non-islands. The local CpG density has been shown to influence the role of methylated cytosines, whereby methylation influences the transcriptional efficacy stronger in high-density islands as compared to non-islands [22]. To ensure that variation in methylation reflect expression levels, the analysis was limited to CpG-sites located within 1500 base pairs up and upstream of the transcriptional start site. Wagner et al. revealed that the relationship between DNA methylation and gene expression is closely related within this region [23].

### Data processing

To quantify methylation levels of CpG sites, raw data were transformed into β-values using the GenomeStudio software (Illumina, San Diego, CA, USA). Further data processing was performed using the R package Illumina Methylation Analyzer (IMA). CpG sites were excluded if located on sex determining chromosomes, covering known SNP loci or if 75 % of subjects exhibited a detection *p*-value greater than 5×10-05.

#### Adjustment of type I and type II probes and removal of batch effects

CpG sites of the Illumina chip belong to type I or type II probes, which significantly differ in distribution and dynamic range of DNA methylation. As this design may bias downstream analyses, methylation data was further adjusted by peak correction and quantile normalization to adjust for probe type differences. The ComBat function [24] was used to adjust for batch effects.

After all steps of data processing procedures were performed, 124 199 probes remained to be included in the subsequent analysis.

### Statistical analysis

All statistical analyses were perfomed using R statistics, version 3.0.2.

‘Hmisc’ package of R statistic was used for discovery group analysis. When sample sizes exceed nine observations, as was the case in our study, this package by default uses algorithm AS 89 and an Edgeworth series approximation to calculate correlation coefficients and *p*-values for Spearman’s rank correlation coefficients. We applied this method of analysis to our data. Percentage change in methylation states and SBP were calculated, measured before and six months after RYGB surgery. These parameters were correlated in using the Spearman’s rank correlation method, resulting in 124 199 individual correlation analyses. *P*-values were adjusted for multiple testing using Bonferroni correction method [25]. Adjusted *p*-values < 0.05 were considered significant. To exclude potential confounding effects of age, BMI and levels of fasting plasma glucose, the same method of analysis was performed, correlating percentile changes in methylation states to these variables. Paired t-tests were performed to evaluate changes in continuous variables independently from each other, e.g. the methylation state of a CpG site before and six months after surgery.

In a second step, we aimed to further investigate the association of changed methylation patterns in candidate CpG sites with EH in a second cohort (verification cohort). For this purpose, binary logistic regressions of EH to the methylation states were performed, accounting for age, BMI, ethnicity and sex as co-variables.

## Results

### Behavior of clinical outcome variables in the discovery group and verification cohort

In the discovery group, comprising patients who underwent bariatric surgery, we initially aimed to identify candidate CpG sites, in which modifications of the epigenetic profile are associated with changes in SBP levels after surgical intervention. As expected, patients reduced their BMI by 24 % six months after surgery. Furthermore, diastolic blood pressure (DBP) and SBP levels were significantly lower after the operation (Table 1).

**Table 1:** Characteristics of subjects included into the study

	Discovery group^a^	Verification cohort	
Parameter		EH	NT
*N*	11	275	264
Men: Women (*n* (%))	7:4	140 (50.9):135 (49.1)	127 (48.1):137 (51.9)
Caucasian: Hispanic (*n* (%))	11:0	132 (48.0):143 (52.0)	97 (36.7):167 (53.3)
Age (years, *n* (%))			
0–19	–	–	1 (0.4)
20–29	1 (9.1)	–	10 (3.8)
30–39	1 (9.1)	7 (2.5)	11 (4.2)
40–49	4 (36.4)	26 (9.5)	38 (14.4)
50–59	3 (27.3)	70 (25.5)	48 (18.2)
60–69	2 (18.2)	73 (26.5)	66 (25.0)
70–79	–	59 (21.5)	51 (19.3)
80–89	–	34 (12.4)	33 (12.5)
90–99	–	5 (1.8)	6 (2.3)
100+	–	1 (0.4)	–
Mean (SD)	46.9 (11.9)	64.9 (13.2)	62.3 (16.4)
Body mass index (BMI)			
Baseline^A^	47.9 (6.9)	29.6 (6.1)	26.9 (4.8)
Post surgery^B^	36.0 (6.7)	–	–
*p*: A vs. B	6.21E–09	–	–
Diastolic blood pressure			
Baseline^A^	91.6 (12.8)	N/A	N/A
Post surgery^B^	82.7 (7.2)	–	–
*p*: A vs. B	1.48E–02	–	–
Systolic blood pressure			
Baseline^A^	144.5 (16.0)	N/A	N/A
Post surgery^B^	125.9 (14.7)	–	–
*p*: A vs. B	6.78E–03	–	–

*Abbreviations*: *EH* essential hypertension, *NT* normotensive, *RYGB* Roux-en-Y gastric bypass, *N/A* not applicable

^a^This group comprises patients who underwent RYGB surgery. Values at baseline and post-surgery are shown as mean values (SD). *P*-values were calculated by means of pairwise t-tests, contrasting values at baseline and six months after RYGB-surgery (*p*: A vs. B). A one-tailed *p*-value <0.05 was considered significant

CpG sites that correlated with SBP in the discovery group were further investigated for an association with EH in the verification cohort. This cohort comprised 539 subjects of Caucasian or Hispanic descent, aged 19 to 101 years and subgrouped into EH or normotensive individuals. While no significant differences with regard to ethnicity were observed in the hypertensive group, Hispanics were overrepresented in the normotensive study arm. In the hypertensive study arm white subjects were older than subjects of Hispanic origin. Furthermore, the individuals showed a significantly higher age and BMI in the hypertensive group as compared to the individuals in the normotensive study arm (Table 1).

### Methylation changes at distinct CpG sites correlate with the change in SBP in obese patients before and six months after RYGB surgery

We performed a genome-wide methylation analysis in obese patients before and 6 months after RYGB surgery and compared the global methylation change with changes in SBP before and after the intervention. In case of 24 CpG sites changes in methylation significantly correlated with the percentile change in SBP six months after RYGB surgery (Table 2). The changed methylation states of these CpG sites did not correlate with age, BMI or DBP, underlining the specificity of the observed associations. Methylation changes of four of these probes correlated also significantly with fasting plasma glucose levels (Table 3). Overall, no significant differences in the methylation of the 24 detected CpG sites were detected before and six months after surgical intervention (Table 2).

**Table 2:** CpG sites significantly correlated with SBP and EH

			Discovery group^a^ (*n*=11)						Verification cohort^b^ (*n*=539)			
			% DNA Methylation (SD)			Spearman’s rank correlation coefficients (SBP)				Binary logistic regressions (EH)		
Gene	Illumina ID	Distance to TSS	Baseline^A^	After surgery^B^	*p* A vs. B	Coef.	*p*	*p* (Bonf.)	% DNA Methylation (SD)	Exp (B)	*p*	*p* (Bonf.)
*MCCC1*	cg00161968	27	5.36 (1.1)	5.9 (1.7)	*ns*	0.93	<10E–16	<10E–16	7.5 (4.3)	–	*ns*	*ns*
*SKOR2*	cg00875989	1500	33.2 (1.7)	33.6 (3.6)	*ns*	(0.92)	<10E–16	<10E–16	37.5 (4.2)	0.92	1.16E–03	2.79E–02
*VGLL3*	cg06251539	375	9.3 (2.4)	8.7 (1.88)	*ns*	0.92	<10E–16	<10E–16	14.5 (3.1)	–	*ns*	*ns*
*WSB1*	cg08706258	125	4.3 (1.1)	4.2 (0.7)	*ns*	0.93	<10E–16	<10E–16	4.8 (0.8)	–	*ns*	*ns*
*EHMT2*	cg09134341	(1332)	91.0 (2.8)	91.8 (1.2)	*ns*	(0.92)	<10E–16	<10E–16	89.3 (1.9)	0.84	9.58E–04	2.30E–02
*TCF12*	cg10146710	1132	2.4 (0.5)	2.4 (0.4)	*ns*	0.92	<10E–16	<10E–16	2.3 (1.0)	–	*ns*	*ns*
*MAP2K4*	cg10596925	(242)	12.1 (3.4)	13.0 (2.0)	*ns*	0.95	<10E–16	<10E–16	10.7 (2.9)	0.94	4.98E–02	*ns*
*FAM54A*	cg10640093	(196)	3.8 (1.0)	4.1 (0.7)	*ns*	0.94	<10E–16	<10E–16	3.0 (0.7)	–	*ns*	*ns*
*SORBS1*	cg12360759	598	7.4 (1.7)	7.4 (1.0)	*ns*	0.92	<10E–16	<10E–16	9.1 (1.3	0.82	6.33E–03	*ns*
*DDX55*	cg15612682	232	6.0 (1.5)	6.1 (0.8)	*ns*	0.93	<10E–16	<10E–16	5.6 (1.0)	–	*ns*	*ns*
*ATXN1L*	cg16076930	380	19.0 (2.8)	20.5 (5.1)	*ns*	0.94	<10E–16	<10E–16	21.1 (2.8)	–	*ns*	*ns*
*EFEMP1*	cg16118212	227	12.7 (4.4)	10.7 (2.9)	*ns*	0.92	<10E–16	<10E–16	17.3 (6.2)	–	*ns*	*ns*
*CRKL*	cg16500810	(197)	1.5 (0.3)	1.6 (0.9)	*ns*	0.92	<10E–16	<10E–16	0.6 (0.6)	–	*ns*	*ns*
*STEAP3*	cg18643762	(211)	2.9 (1.0)	3.4 (1.0)	*ns*	(0.93)	<10E–16	<10E–16	1.7 (1.3)	–	*ns*	*ns*
*CPEB4*	cg20841073	648	10.7 (2.2)	10.3 (2.7)	*ns*	0.93	<10E–16	<10E–16	5.2 (2.0)	0.85	2.37E–03	*ns*
*RHOBTB2*	cg21344124	302	3.2 (0.7)	2.8 (1.1)	*ns*	0.92	<10E–16	<10E–16	2.2 (0.7)	1.34	1.96E–02	*ns*
*ATXN1*	cg21996137	(185)	2.0 (0.7)	1.8 (0.6)	*ns*	0.92	<10E–16	<10E–16	1.6 (0.7)	1.42	1.60E–02	*ns*
*TSSK1B*	cg22011370	525	89.9 (2.6)	89.9 (2.9)	*ns*	(0.95)	<10E–16	<10E–16	90.6 (3.3)	–	*ns*	*ns*
*SPPL2A*	cg22295383	(104)	5.2 (1.3)	4.9 (1.1)	*ns*	0.94	<10E–16	<10E–16	4.2 (1.3)	–	*ns*	*ns*
*BHLHA9*	cg23945265	1003	45.4 (4.2)	46.9 (3.2)	*ns*	(0.92)	<10E–16	<10E–16	45.7 (5.2)	0.96	2.58E–02	*ns*
*CHCHD5*	cg25521086	(58)	4.0 (0.8)	3.8 (0.7)	*ns*	0.95	<10E–16	<10E–16	3.1 (1.2)	–	*ns*	*ns*
*NRTN*	cg25544164	342	99.0 (0.3)	9.1 (0.2)	*ns*	0.94	<10E–16	<10E–16	99.1 (0.5)	–	*ns*	*ns*

*Abbreviations*: *EH* essential hypertension, *Exp(B)* log odds ratio, *ns* not significant, *RYGB* Roux-en-Y gastric bypass, *SBP* systolic blood pressure, *SD* standard deviation, *TSS* transcription start site

^a^This group comprises patients who underwent RYGB surgery. ^b^Results were derived from binary logistic regressions of EH (yes/no) to % CpG site methylation, age, BMI, ethnicity and sex. Log odds ratios and *p*-values refer to the CpG site in focus. *P*-values for DNA methylation levels were calculated by paired t-tests, contrasting methylation levels at baseline and six months after RYGB-surgery (*p*: A vs. B). GWAS results for SBP were calculated by genome-wide analyses of Spearman's rank correlation coefficients using algorithm AS 89 and an Edgeworth series approximation. Unless specified as SD, parenthesis “()” implies negative numbers

**Table 3:** CpG site associations to age, BMI, DBP and glucose

Discovery cohort^a^ (*n* = 11)					
		Spearman’s rank correlation coefficients			
Gene	Illumina ID	*p* (Age)	*p* (BMI)	*p* (DBP)	*p* (Glucose)
*MCCC1*	cg00161968	ns	ns	ns	ns
*SKOR2*	cg00875989	ns	ns	ns	ns
*VGLL3*	cg06251539	ns	ns	ns	ns
*WSB1*	cg08706258	ns	ns	ns	ns
*EHMT2*	cg09134341	ns	ns	ns	ns
*TCF12*	cg10146710	ns	ns	ns	2.81E–02
*MAP2K4*	cg10596925	ns	ns	ns	ns
*FAM54A*	cg10640093	ns	ns	ns	ns
*SORBS1*	cg12360759	ns	ns	ns	ns
*DDX55*	cg15612682	ns	ns	ns	ns
*ATXN1L*	cg16076930	ns	ns	ns	ns
*EFEMP1*	cg16118212	ns	ns	ns	ns
*CRKL*	cg16500810	ns	ns	ns	2.08E–02
*STEAP3*	cg18643762	ns	ns	ns	ns
*CPEB4*	cg20841073	ns	ns	ns	ns
*RHOBTB2*	cg21344124	ns	ns	ns	ns
*ATXN1*	cg21996137	ns	ns	ns	ns
*TSSK1B*	cg22011370	ns	ns	ns	4.04E–02
*SPPL2A*	cg22295383	ns	ns	ns	ns
*BHLHA9*	cg23945265	ns	ns	ns	4.78E–02
*CHCHD5*	cg25521086	ns	ns	ns	ns
*NRTN*	cg25544164	ns	ns	ns	ns

*Abbreviations*: *DB* diastolic blood pressure, *ns*, not significant, *RYGB*, Roux-en-Y gastric bypass, *SBP* systolic blood pressure

^a^Cohort comprises patients who underwent RYGB surgery. *P*-values were derived by analyses of Spearman’s rank correlation coefficients using algorithm AS 89 and an Edgeworth series approximation. For the variables DBP, BMI and fasting plasma glucose, percent change in methylation states were correlated with percent change in these variables, measured before and six months after RYGB surgery. For the variable age, percent change in methylation states measured before and six months after RYGB surgery was correlated with age.

### The methylation of two CpG sites that correlated with SBP levels in the RYGB cohort are differentially methylated in the confirmative cohort showing EH

Two candidate CpG sites (cg00875989; cg09134341) that we found to be associated with SBP in the RYGB cohort, were significantly hypomethylated in EH (Table 2). These sites are located in the promoter regions of the genes Euchromatic histone-lysine n-methyltransferase 2 (EHMT2) and ski family transcriptional corepressor 2 (SKOR2), respectively. The results were confirmed in the verification cohort by comparing methylation levels in normotensives and EH by binary logistic regression analysis and were independent of age, BMI, ethnicity and sex (Table 2).

## Discussion

By genome-wide investigating the DNA methylation profile in whole blood of RYGB patients we identified two novel CpG sites that were associated with SBP levels and EH. Our findings show that increased methylation of these probes correlate strongly with significant reduction in SBP levels observed after RYGB surgery. In agreement with this, EH is associated with significantly lower methylation levels of the identified CpG sites compared to normotensive subjects. These results were derived from two independent study populations encompassing healthy, obese, young, middle-aged and elderly subjects, respectively. The observations suggest that the identified epigenetic risk sites are associated with hypertension and cardiovascular disease across different ages and weight groups.

To our knowledge, this is the first study investigating epigenome-wide associations of whole blood methylation patterns and SBP levels in a longitudinal manner in a RYGB cohort. By observing RYGB patients before and after intervention, we were able to take the dynamic quality of BP and associated methylation changes into account. Thus, our study allows more specifically elucidating BP associated methylation changes as compared to e.g. a case-control study set up, where the likelihood of confounding due to interindividual variance and e.g. (long-time) HT induced secondary methylation changes is much higher. By choosing a genome-wide study approach using the Illumina 450 k methylation chip, it was possible to comprehensively investigate 124 199 CpG sites with regard to changes in methylation patterns in a hypothesis-free and thereby unbiased manner. The two CpG sites did not change significantly in their methylation state after RYGB surgery, providing further grist to the mill that their association to SBP is not limited exclusively to this intervention but rather attributable to the general population.

**Fig. 1:**
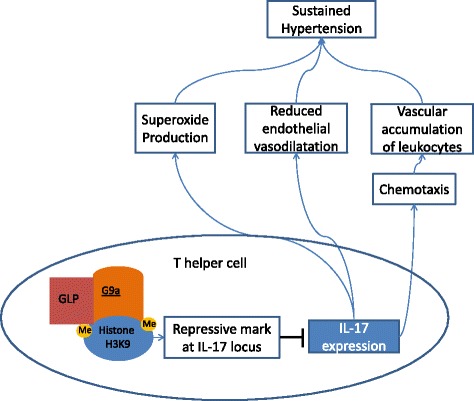
The methyltransferase G9a in cooperation with its homologue G9a-like protein (GLP) is responsible for the majority of dimethylation of H3K9me2. Cells that lack G9a (or GLP) display a drastic reduction in this modification [26]. H3K9me2 acts as a major repressive histone methylation mark in euchromatin, that affects also the interleukin 17 gene locus, resulting in inhibition of IL-17 expression [27]. Harrison et al. demonstrated that mice lacking IL-17 do not develop sustained hypertension after treatment with angiotensin II. In contrast to wild-type mice, IL-17−/− mice do not exhibit an increase in superoxide production and there is no reduction in endothelial-dependent vasodilatation. Furthermore, there was a reduction in vascular accumulation of leukocytes in IL-17−/− mice, probably due to a reduction of chemotaxis normally induced by IL-17 [35]

Euchromatic histone-lysine n-methyltransferase 2 (EHMT2; also termed G9a) is a protein lysine histone methyltransferase (PKMT), encoded by *EHMT2* and expressed in high levels in peripheral blood leukocytes [26]. G9a is responsible for the majority of dimethylation of histone H3 at lysine 9 (H3K9me2), a major repressive histone methylation mark in euchromatin [27]. Cells lacking G9a display a drastic reduction in this modification [27]. Lehnertz et al. identified a critical role for G9a in T helper cell differentiation in vitro and in vivo. Notably, expression of interleukin 17 (IL-17) was dysregulated in T helper cells in the absence of G9a or in the presence of a specific G9a methyltransferase inhibitor, which is associated with a loss of H3K9me2 at the IL-17 locus. G9a is thus a critical component in the inhibition of IL-17 expression [28]. Several recent studies confirmed the concept that cytokines produced by T cells and other inflammatory cells contribute to hypertension [29–35]. Harrison et al. identified IL-17 as a novel pro-inflammatory cytokine that contributes to the development of hypertension. Notably, Harrison et al. found that mice lacking IL-17 do not sustain hypertension after treatment with the hypertensive stimulus angiotensin II [36]. Furthermore, in contrast to the effects seen in wild-type mice, IL-17−/− mice do not exhibit an increase in superoxide production and there is no reduction in endothelial-dependent vasodilatation after treatment with angiotensin II [36]. IL-17 further promotes chemotaxis of other proinflammatory cells by stimulating release of chemokines [37, 38]. In agreement with this, Harrison et al. also noted a reduction in vascular accumulation of leukocytes in IL-17−/− mice treated with angiotensin II [36]. Thus, IL-17 may influence the vascular pathophysiology of hypertension by its direct effects - increasing superoxide production and reducing endothelial-dependent vasodilatation - and by guiding other pro-inflammatory cells to the perivascular tissue. Further studies are needed to confirm the role of IL-17 in human EH, and to investigate whether the observed methylation changes in G9a reflect gene expression levels, H3K9me2 activity and IL-17 expression levels. The global role of G9a leaves of course space for additional regulatory functions in HT by, for example, incorporating also effects on the induction of Th2 lineage-specific cytokines interleukin 4, interleukin 5 and interleukin 14 [27] and the cellular response to stress by inhibition of interleukin 6 and interleukin 8 transcription [39]. Nevertheless, in light of these recent scientific advances in the study of hypertension, we suggest that G9a putatively contributes to the pathophysiology of EH by influencing IL-17 expression (Fig. 1).

SKI family transcriptional co-repressor 2 (SKOR2), also termed functional SMAD-suppressing 235 element on chromosome 18 (FUSSEL18) [40] or Corl2 [41], was first described by Arndt et al. and 236 identified as a novel homolog to the SKI family of transcriptional co-repressors [40]. While little is known about its role in human leukocytes, cancer studies have identified this protein as a potential tumor suppressor in head and neck squamous cell carcinomas [42]. Further research is needed before any speculation as to the functional relevance of SKOR2 in EH can be made.

Our study is limited by the fact that BP was measured only once in a sitting position in the discovery cohort, which putatively causes a higher variance of the outcome variable than expected with repeated and averaged measurements. We took the most important confounders available, such as age, gender, BMI and ethnicity, into consideration, on the association analysis between methylation and EH. However, potential confounding factors e.g. dietary patterns, comorbidities or intake of any antihypertensive medication may be able to induce changes in methylation patterns as well. Furthermore, our study pertained to promoter associated CpG sites in DNA methylation in whole blood largely consisting of leukocytes, which are composed of different cell populations with unique epigenetic profiles [43]. Studies indicate that changes in leukocyte composition may induce a significant variability in the DNA methylation pattern observed in the peripheral blood [43]. Koestler et al. provided a list of the top 50 differentially methylated CpG sites observed in six important leukocyte subtypes [44]. None of the newly detected CpG sites belonged to the described group of CpG sites, which supports the reliability of our results. Furthermore, we confirmed our findings in more than 539 individuals in a case-control study, making confounding effects on our results due to changes in leukocyte composition unlikely.

## Conclusions

In summary we were able to identify two novel CpG loci associated with EH that have not yet been described in context to HT. Our findings may contribute to a further understanding of the epigenetic regulatory mechanisms behind the development of this disease. Based on our findings we suggest that EHMT2 may contribute to the vascular pathophysiology of EH by influencing IL-17 expression in T helper cells. Further studies are needed to confirm the role of IL-17 in human EH, and to investigate whether the observed methylation changes in G9a reflect gene expression levels, H3K9me2 activity and IL-17 expression levels. The global role of G9a leaves space for additional regulatory functions in HT.
